# Social Connectedness and Communication Technology Among Paid and Unpaid Caregivers of Middle-Aged and Older Adults

**DOI:** 10.1093/geroni/igab046.3318

**Published:** 2021-12-17

**Authors:** Shinduk Lee, Marcia Ory, Deborah Vollmer Dahlke, Matthew Smith

**Affiliations:** 1 University of Utah College of Nursing, Utah, United States; 2 Texas A&M University, College Station, Texas, United States; 3 DVD Associates, LLC, DVD Associates, LLC, Texas, United States; 4 Texas A&M School of Public Health, College Station, Texas, United States

## Abstract

Objective: To examine differential relationships between communication technology use and social connectedness among paid and unpaid caregivers providing care for middle-aged and older adults. Methods: This nationwide study utilized a cross-sectional Qualtrics panel survey from caregivers, ages 50 years and older (n=504) of middle-aged and older adults. About 17% were paid (n=86) and 83% (n=418) were unpaid caregivers. Primary outcomes were caregivers’ sense of belonging to their local community and social bonds. The primary predictor of interest was caregivers’ use of communication technology (texting/communication applications). A multivariable regression analysis was performed to predict each outcome based on communication technology use, separately among paid and unpaid caregivers. Multivariable regression analyses were repeated after including the caregivers’ payment status (paid/unpaid) and the interaction term between the caregivers’ payment status and the use of communication technology. The models were adjusted for caregivers’ age, education, financial status, place of residence, and total weekly hours of caregiving. Results: Use of communication technology had a statistically-significant positive association with sense of belonging only among paid caregivers (b=25.8, p=.005). The relationship between use of communication technology and sense of belonging was significantly different between paid and unpaid caregivers (p=.005). Use of communication technology was significantly associated with social bonds only among unpaid caregivers (b=0.4, p<.001). There was no statistically-significant differential association between communication technology use and social bonds. Conclusion: Communication technology may play differential roles in linking paid and unpaid caregivers with their community and interpersonal groups. Additional efforts should examine mechanisms that provide meaningful caregiver support.

